# Part-solid pulmonary nodule phantoms with realistic morphology and densities by stereolithography-based 3D-printing: from design to validation

**DOI:** 10.1186/s41747-025-00644-4

**Published:** 2025-11-04

**Authors:** Louise D’hondt, Dimitri Buytaert, Pieter-Jan Kellens, Annemiek Snoeckx, Klaus Bacher

**Affiliations:** 1 Department of Human Structure and Repair, Faculty of Medicine and Health Sciences, Ghent University, Ghent, Belgium; 2 Faculty of Medicine, University of Antwerp, Wilrijk, Belgium; 3 Department of Radiology, Antwerp University Hospital, Edegem, Belgium

**Keywords:** Lung neoplasms, Multiple pulmonary nodules, Phantoms (imaging), Stereolithography, Tomography (X-ray computed)

## Abstract

**Background:**

Oversimplified pulmonary nodule phantoms limit the clinical translation of computed tomography (CT) research. Therefore, we manufactured and preliminarily validated patient-realistic part-solid nodule models with heterogeneous radiodensities using a stereolithography apparatus (SLA) benchtop three-dimensional (3D) printing.

**Materials and methods:**

Patient-derived CT images were utilised upon Ethics Committee approval to determine part-solid nodule HU ranges and morphologies. To lower the density of the applied resin (Formlabs Clear V4), we designed variable 3D-beams (340, 510, or 680 µm) into lattice structures with variable gap thicknesses (from 680 to 2,040 µm). These lattice structures were merged with patient-derived nodule segmentations. The 3D-prints were incorporated in a Lungman phantom and evaluated using low-dose CT imaging. A multicentre, single-blinded reader study, involving seventeen radiologists, assessed whether 3D-printed nodules were distinguishable from real part-solid nodules using *χ*^2^ goodness-of-fit testing.

**Results:**

Through varying combinations of material thicknesses and void sizes, we reproduced multiple target radiodensities of clinical nodules and successfully manufactured pulmonary nodule phantoms consisting of three different ground-glass components around a solidly printed core. There was significant evidence (*χ*^2^ = 136.13; *p* = 1.864e-31; 5% confidence level) against readers reliably distinguishing patient nodules from our 3D-printed models. Average accuracy across all radiologists was 53.5%. Moreover, 47.5% of the 3D-printed nodules were incorrectly classified as real nodules.

**Conclusion:**

Our SLA 3D-printing workflow produces patient-realistic part-solid pulmonary nodules that are more cost-effective than commercially available counterparts. This methodology could provide customisable ground truth phantom models for CT imaging studies, including software validation, acquisition and reconstruction parameter optimisation and/or image quality evaluation.

**Relevance statement:**

This study marks the first successful application of SLA 3D-printing to manufacture part-solid pulmonary nodule phantoms, incorporating multiple radiodensities and mimicking patient-realistic morphologies. Our developed methodology offers potential to 3D-printed phantoms with higher degrees of customisation and adaptation to research-specific objectives in CT imaging compared to commercially available standardised phantoms.

**Key Points:**

- Part-solid pulmonary nodule phantoms with patient-realistic morphologies and multiple radiodensities were manufactured using benchtop SLA 3D-printing.
- Clinical relevance of our 3D-printed nodules is demonstrated and statistically substantiated in a multicentre, single-blinded reader study including seventeen reading radiologists.
- Our methodology renders pulmonary nodule models that overcome limitations of generic, standardised, commercially available phantoms, often lacking complexity and realism.
- Manufactured nodule phantoms can provide an absolute ground truth for software training and validation, CT protocol optimisation and (image) quality assurance.
- Our modified 3D-printing method is readily available to other groups and can be customised to specific research applications.

**Graphical Abstract:**

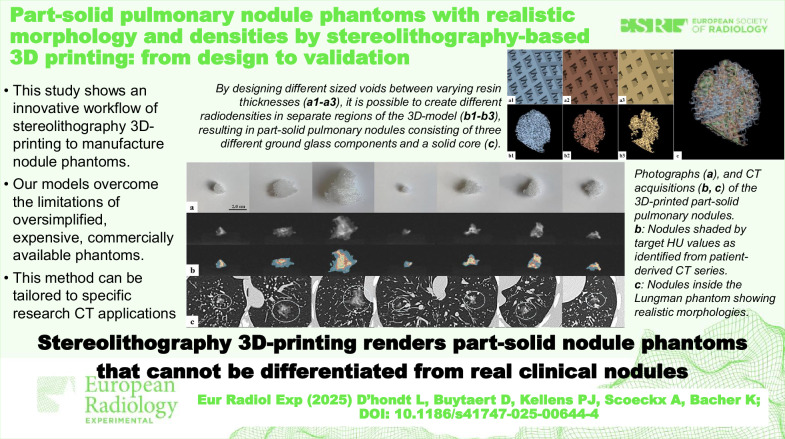

## Background

Computed tomography (CT) research applies phantom models to objectively assess and optimise procedures in terms of image quality and dosimetric accuracy without patient exposure [1–9]. However, currently available phantoms mostly oversimplify intricate human morphologies, limiting clinical translation of results [1, 2, 5, 8, 10–13]. Consequently, different research groups have manufactured personalised anthropomorphic phantoms as a tool to replace and/or complement generic phantoms for CT applications [3–8, 11–18]. Recently, additive manufacturing, also known as 3D-printing, has become more widely available, allowing cost-effective and automated fabrication of customised phantoms [4, 6, 9, 10, 14, 15, 17, 19–21].

Although 3D-printing allows fast and relatively inexpensive manufacturing of custom phantoms, these models often lack anatomical details and must exhibit equivalent radiological attenuation as human tissues in terms of HU values [3, 4, 10, 12, 14–16, 18, 22–26]. The attenuation of x-rays depends on the physical density of the exposed tissues, leading to a grayscale in reconstructed CT images [27, 28]. However, the linear attenuation coefficients, intrinsic to the HU values, are often undisclosed or materials with the same required range of radiation attenuation characteristics are not readily available [11, 12, 14, 18, 19, 24, 26]. Furthermore, gradients of HU values within the same human structure can be challenging to simulate accurately in a 3D-printed phantom [3].

Several studies explored various methods to create phantoms with diverse attenuating properties for CT [8, 12, 16, 22–26, 29–32]. The most common approach involves fused deposition modelling (FDM), where a melted plastic filament is extruded layer-wise, often in a grid pattern [3, 4, 10, 14, 17, 19, 22–25, 30, 32]. FDM produces inhomogeneous 3D-prints due to air gaps, inherently enabling density variation via infill modification [11, 14, 17, 24, 31]. In contrast, stereolithography apparatus (SLA) employs an ultraviolet laser to cure layers in liquid photopolymer (resins), producing gap-free structures with higher resolution and accuracy than FDM [11, 14, 17, 31]. However, no methods have been developed to simulate variable tissue densities as observed on CT images, as SLA is typically limited to single material, fully dense printing [17].

Part-solid pulmonary nodules are key examples of structures with intricate morphologies and varieties of radiodensities. The primary differential diagnosis for these nodules is early lung cancer. With increased CT-based detection and the advent of lung cancer screening, nodule characterisation has become a widely researched topic [4, 13, 33–42]. However, studies using generic phantoms report oversimplification, limiting clinical relevance [34, 36–39, 41, 42]. Realistic 3D-printed anthropomorphic models could provide a reliable ground truth that is relatively inexpensive and quickly available compared to the generic, commercial counterparts [3, 4, 13, 33]. Pure ground-glass opacities and solid nodules, consisting of one density, have been successfully manufactured mostly with FDM 3D-printing to match patient-realistic cases [3, 4, 13, 18, 33, 35]. However, prior studies were not completely able to manufacture 3D-printed part-solid nodules that embody multiple radiodensities in the design, as well as an overall replication and validation of humanlike morphology [3].

We used in-house available 3D-printing to create part-solid pulmonary nodule models for CT research that adequately represent clinical reality in terms of morphology and radiodensities. We developed a custom SLA-printing methodology, enabling the achievement of radiodensities beyond the intrinsic resin density and mimicking complex morphologies of nodules. The 3D-printed nodules were additionally validated through a single-blinded reader study, evaluating their visual and structural fidelity compared to real nodules. To our knowledge, this is the first study to successfully manufacture nodule phantoms with SLA-technology that capture both complex morphological features as well as varying HU heterogeneities within different regions of the nodule.

## Materials and methods

**Fig. 1:**
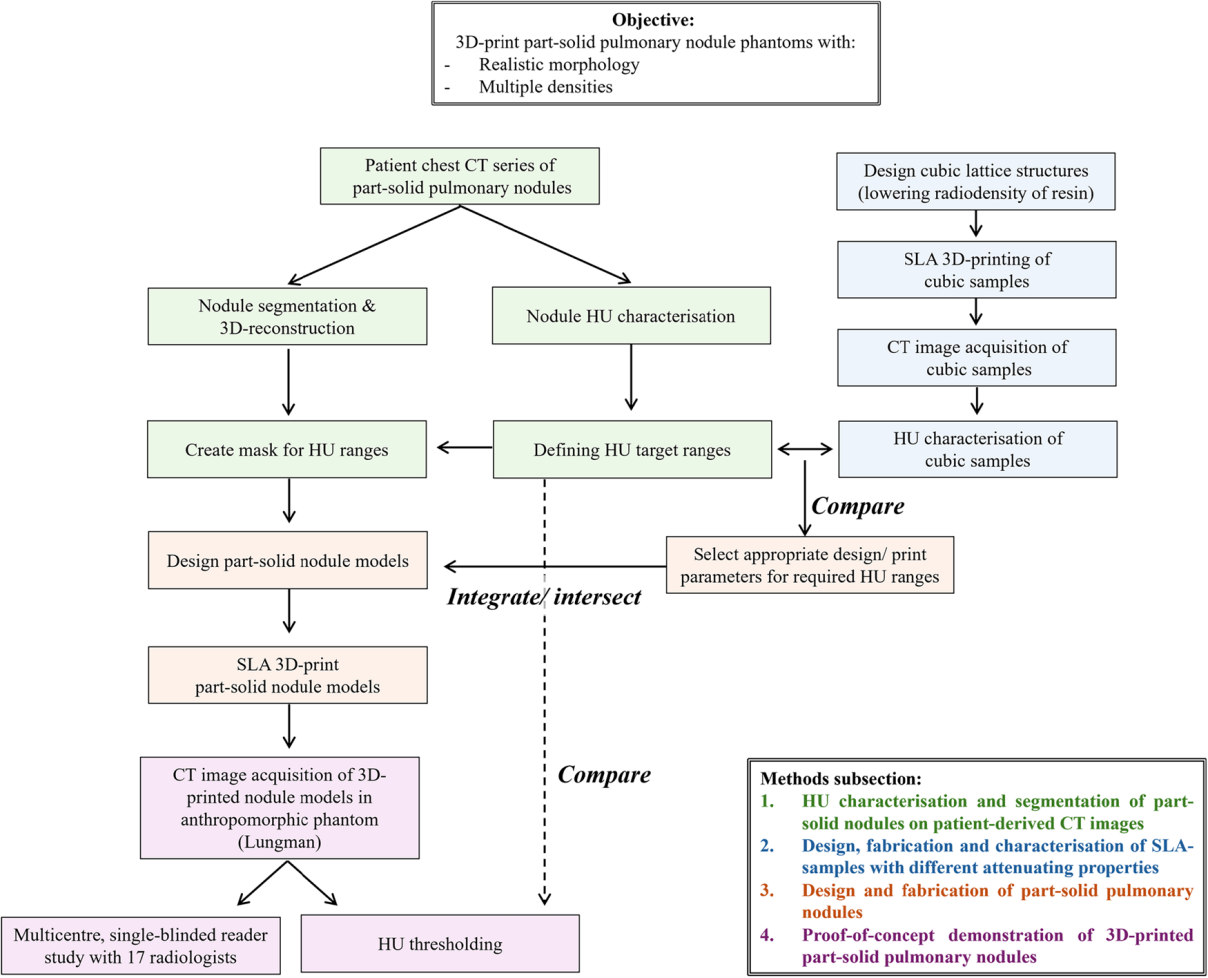
Schematic flowchart of the study set-up. CT, Computed tomography; SLA, Stereolithography apparatus

Figure 1 summarises the study set-up detailed below.

### HU characterisation and segmentation of part-solid nodules on patient-derived CT images

**Fig. 2:**
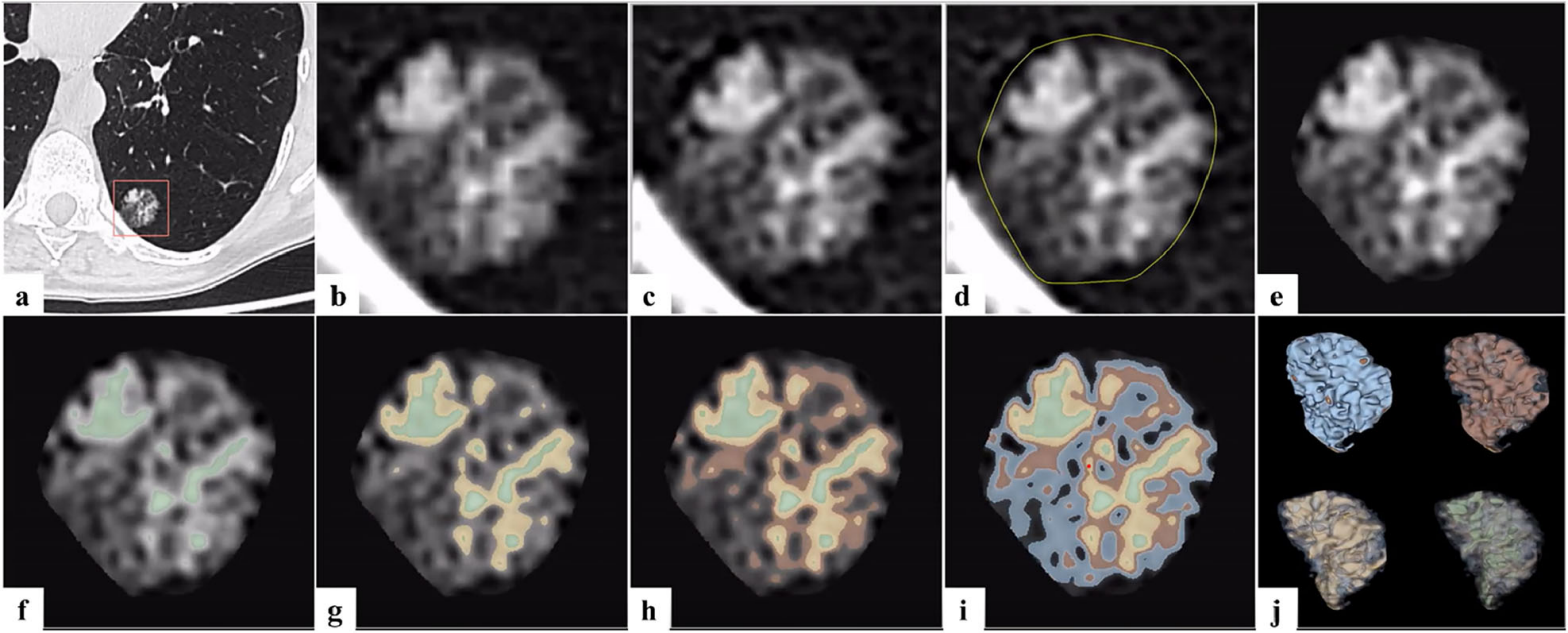
Nodule segmentation process. Example of the stepwise segmentation and thresholding using 3D Slicer on a patient’s chest computed tomography scan of a part-solid pulmonary nodule. **a** Define the cropping region of interest around the nodule (orange square). **b** Crop out the nodule and save as a new volume. **c** Resample the cropped volume to voxels with an isotropic spacing of 0.1 × 0.1 × 0.1 mm^3^. **d** Segment the nodule (remove background outside of the yellow circle). **e** Create a masked volume containing only the segmented nodule. **f**−**i** Segment masked volume by HU thresholding: green (> 0 HU); yellow (0; -300 HU); red (-300; -500 HU), and blue (-500; -750 HU); **j** HU threshold segmentations converted to a three-dimensional mesh

Six anonymised chest CT scans, from patients with part-solid pulmonary nodules, were obtained from the Radiology Department of Antwerp University Hospital, with ethical committee approval (Ethics Committee Approval Number: EC/PM/eva/2024.048). The retrospective CT series was acquired and reconstructed with different parameters (Supplementary Table S1). Figure 2 details the segmentation and 3D-reconstruction of the part-solid pulmonary nodules on the patient-derived CT images. Using the 3D Slicer software (v 5.6.1), we segmented and thresholded four different ranges of HU values on the patient nodule CT voxels, creating an overlay for each range. These four HU ranges were based on more definite HU measurements on the CT scans recorded in Fiji, an open-source image analysis software [43]. A circular region of interest was placed in the distinctive solid core and ground-glass component of each nodule. Three measurements were taken in each region, and standard deviations were calculated and reported (Table S1). The minimum measured HU on patient scans (-750 HU) was set as the lower limit of the targeted HU range. Three chosen HU ranges were defined and segmented as regions of ground-glass opacities ((0; -300 HU), (-300; -500 HU), (-500; -750 HU)) that gradually merge into the solid core (> 0 HU) (see Fig. 2).

### Design, fabrication, and characterisation of SLA samples with different attenuating properties

**Fig. 3:**
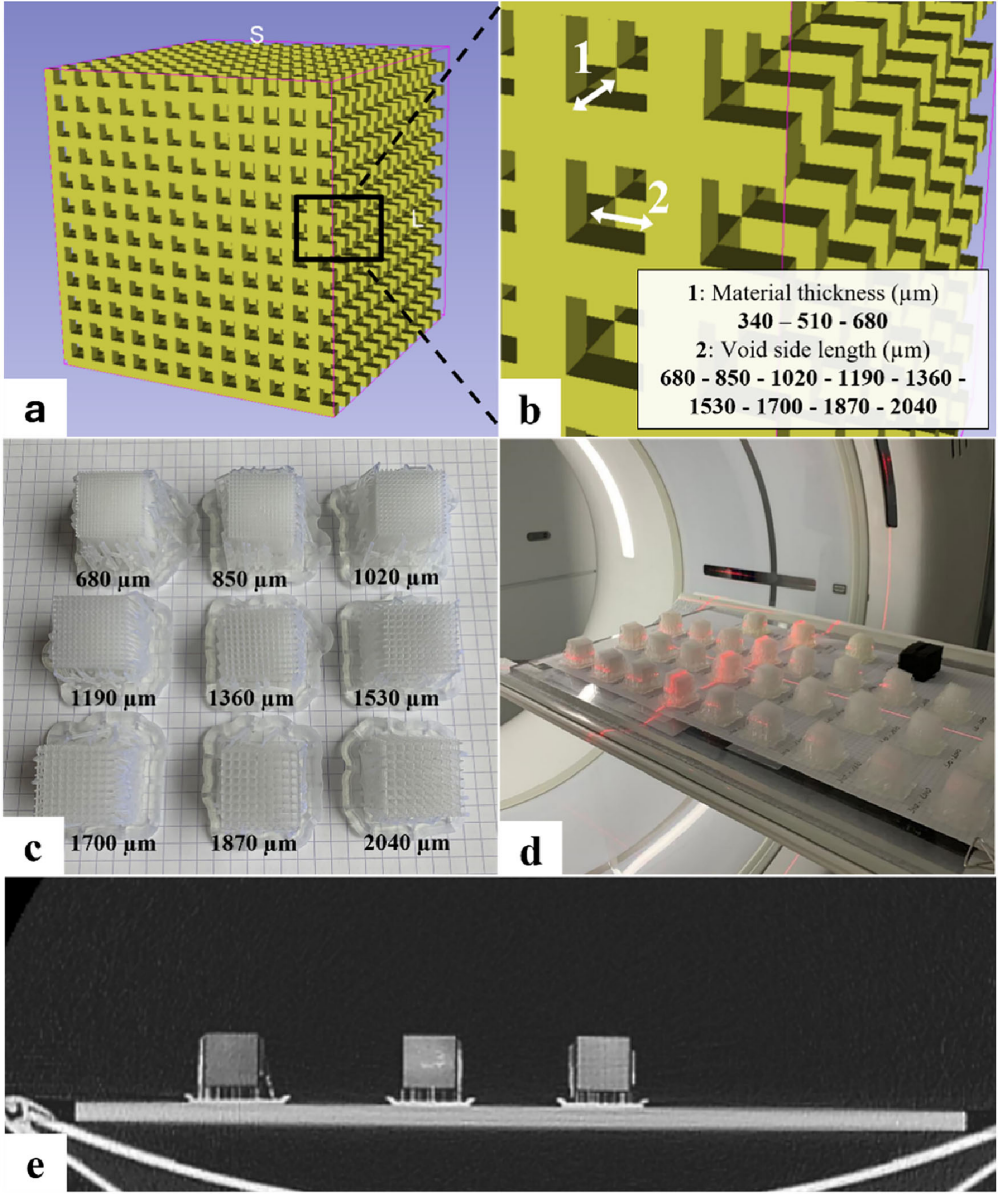
Design and characterisation of cubic SLA-printed samples. **a** Designed cubic samples with a lattice structure. **b** Magnified view of the 3D-beams of variable thickness (1: material thickness) placed into a 3D-grid of variable spacing (2: Void side length). **c** Picture of cubic samples printed with a material thickness of 340 µm and varying gaps. **d** Image acquisition of the 3D-printed samples on a 256-slice CT scanner (GE Healthcare Revolution). **e** Example of a CT image slice used for HU characterisation. 3D, Three-dimensional; CT, Computed tomography

To achieve HU values lower than the characteristic value of the used SLA print material, we used the partial volume effect, including air in the bulk print, adopting a technique similar to the polymer jetting approach from Leary et al [12]. Cubic samples with a lattice structure of varying material and gap thickness were designed using OpenSCAD, a scripting-based software to create 3D computer-aided design models [44]. Beams of variable thickness were placed into a grid of variable spacing and merged together into a single 3D-cube (Fig. 3). The variable beam thicknesses were defined as multiples of the used 3D-printer’s spot size resolution (85 µm). Gaps or voids in between the grid were manually designed with a length that is a multiple of the minimum spot resolution, ranging from 680 µm up to 2,040 µm, resulting in a total of 18 cubic test samples. All 3D-prints were manufactured by a Formlabs Form 3 printer (Somerville, MA, USA), using a resin with a density of 1.17 g/cm³ (Formlabs Clear V4).

The printed cubes were subsequently scanned with a 256-slice GE Healthcare Revolution CT. Parameters of the thorax protocol were matched to those of the patient's CT images. Image acquisitions were made with tube potentials of 100, 120, and 140 kV at CT dose index values (CTDI_vol_) of 1.50 and 0.20 mGy. All acquisitions were reconstructed with combinations of three reconstruction algorithms (filtered back projection, iterative reconstruction (IR), and deep-learning image reconstruction (DLIR)) and two reconstruction kernels (hard and smooth). On all CT series, the mean HU and standard deviation were measured in Fiji by placing a circular region of interest on three distinct slices in every cube, at a sufficient distance from the edge of the sample.

### Design and fabrication of part-solid pulmonary nodules

**Fig. 4:**
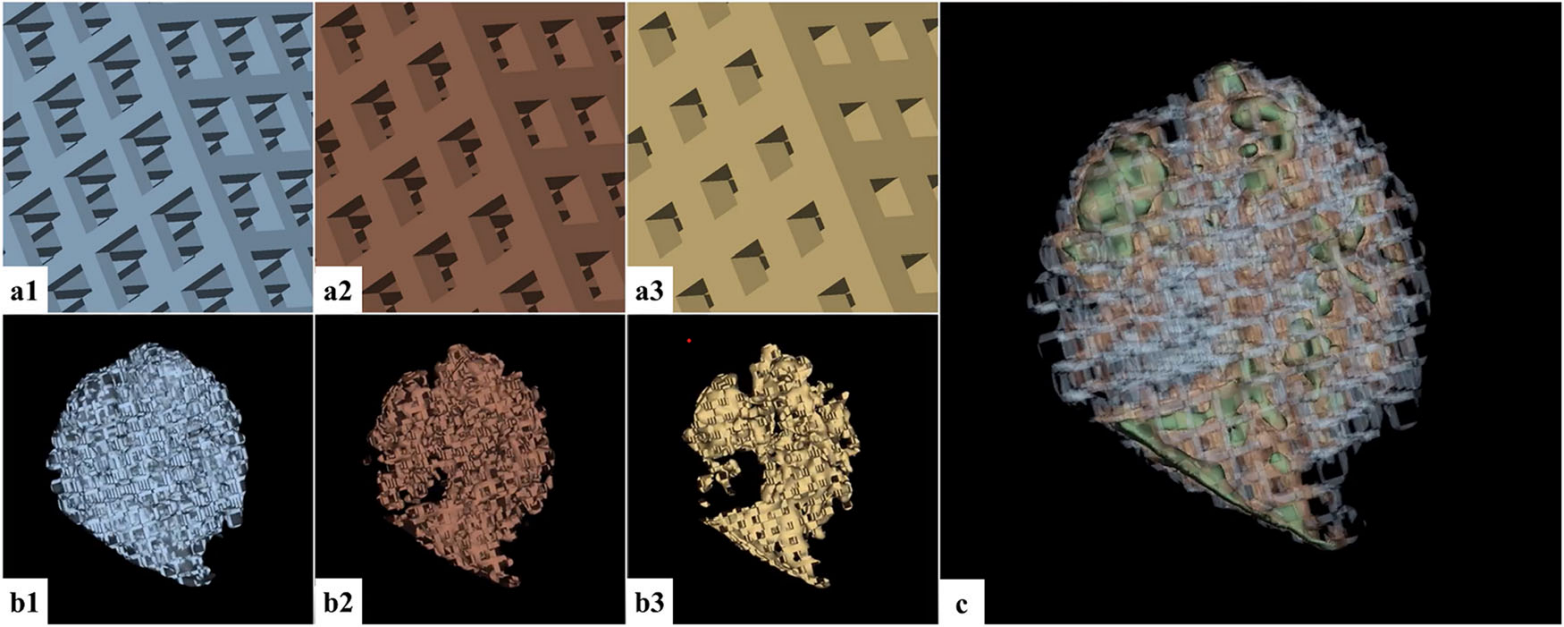
Incorporation of density information in the segmented nodules. Example of the intersection of the selected lattice structures, based on the desired HU value, with segmented HU regions from the patient part-solid nodule. (**a1**, **b1**) Intersection of a density cube with material thickness of 340 µm and holes of 1,020 µm, and part of the nodule that corresponds to a HU range of -500 to -750 HU. (**a2**, **b2**) Intersection of a density cube with material thickness of 510 µm and holes of 850 µm, and part of the nodule that corresponds to a HU range of -300 to -500 HU. (**a3**, **b3**) Intersection of a density cube with material thickness of 680 µm and holes of 680 µm, and part of the nodule that corresponds to a HU range of 0 to -300 HU. **c** Eventually, a Boolean union of the three intersected meshes, combined with the solid nodule mesh (> 0 HU), is merged into a single three-dimensional mesh ready to be printed. The Boolean operations were conducted using Autodesk Netfabb

The HU values determined in prior radiodensity analysis of the patient-specific part-solid nodules (Methods section: “HU characterisation and segmentation of part-solid nodules on patient-derived CT images”) were compared and matched with the HU range achieved in the printed cubic samples. The most suitable 3D-printing designs were selected to mimic nodule radiation attenuation properties. The corresponding designs were introduced in the part of the mesh-file of the segmentation as detailed in Fig. 4. To model the solid part of the nodule, we designed a solid core that is 3D-printed in the resin in a fully dense, gap-free manner. For each segmented HU range mesh of the subsolid part of the patient nodule, a Boolean intersection with the corresponding HU density cube mesh was performed. Ultimately, a Boolean union operation was performed to join the three previous Boolean intersection meshes with the mesh of the solid part of the nodule, resulting in one single 3D-model ready to be printed for each patient nodule. The Boolean operations were conducted using Autodesk Netfabb [45].

The 3D-prints were washed with isopropyl alcohol for 15 min and subsequently cleared from uncured resin stuck in the lattice holes using an air compressor. Afterwards, the prints were cured using ultraviolet light for 20 min at 30 °C. Support structures were then removed. No additional manual post-processing (such as sanding or smoothing) was performed, as the printed lattice was indistinguishable from support contact points.

### Proof-of-concept demonstration of 3D-printed part-solid pulmonary nodules

The 3D-printed nodules were preliminarily validated through a two-centre single-blinded reader study, using ViewDEX software [46]. The goal was to assess whether radiologists could reliably distinguish clinical pulmonary nodules from the 3D-printed models, scanned in a chest phantom. Eligible radiologists affiliated with Ghent University Hospital or Antwerp University Hospital were selected based on training or expertise in chest imaging and experience with reading (part-solid) pulmonary nodules on thoracic oncology images. Radiologists with different levels of aforementioned experience were selected in order to approximate clinical practice and avoid bias in selecting the reader sample [47]. In total, seventeen radiologists (5 senior radiologists with 11 to 34 years of experience and 12 residents in training with up to 5 years of experience) participated. The printed nodules were randomly affixed and imaged inside the anthropomorphic Lungman phantom (Multipurpose chest phantom N1, Kyoto Kaguku Inc. [48]) with the GE Healthcare Revolution CT scanner using a clinical low-dose thorax protocol.

Two distinct reconstructed CT series (DLIR at 0.625-mm slice thickness and IR at 1.25-mm slice thickness) alongside the patient-derived CT series assemble a dataset of 21 unique acquisitions of part-solid nodules. Each series was presented to the readers three times in randomised order using lung window settings (width 1500; level -600). For each series, readers were asked to specify whether the presented image displayed a real part-solid pulmonary nodule or a 3D-printed model of a part-solid pulmonary nodule. Additionally, for each series, the readers were prompted to rate their level of confidence in their assessment, using a defined 5-point Likert scale (5 = completely confident; 4 = fairly confident; 3 = somewhat confident; 2 = slightly confident; 1 = not at all confident). For each nodule within the full CT scan, only the slices containing the nodule were displayed and using 3D Slicer, a masked volume was created containing only the nodule while blanking out any other regions to exclude bias in scoring based on the difference in background between patient images (lung parenchyma) against phantom images (air). Confusion matrices with the numbers of true/false positives and true/false negatives were produced, and the associated error rates and overall accuracies were calculated. 95% confidence intervals for the accuracy and error rates were determined via the bootstrapping method based on 5.000 bootstrap replicates [49, 50] using the “R” package ‘boot’ (v1.3-31). To evaluate inter-reader agreement, Conger’s *κ* coefficient for multiple raters [51–53] was calculated using the “R” package ‘irr’ (v0.84.1). A *χ*^2^ goodness-of-fit test assessed whether radiologists were, on a 5% significance level, able to differentiate between real and 3D-printed part-solid pulmonary nodules or not.

## Results

### HU characterisation and segmentation of part-solid nodules on patient-derived CT images

The CT series of six different patients (seven different part-solid nodules) showed large differences in both shape and size of the nodules as well as in HU values. Specific information regarding the used patient images and the measurement of the HU distribution per patient can be found in the supplementary material (Table S1). Summarised across all patients’ average HU, the higher radiodensity in the centroid of the tumour ranged from 52 to 256 HU (minimum -247, maximum 577), while in the outer subsolid components of the nodules, HU ranged from -635 to -375 (minimum -750, maximum -256).

### Design, fabrication, and characterisation of SLA samples with different attenuating properties

**Fig. 5:**
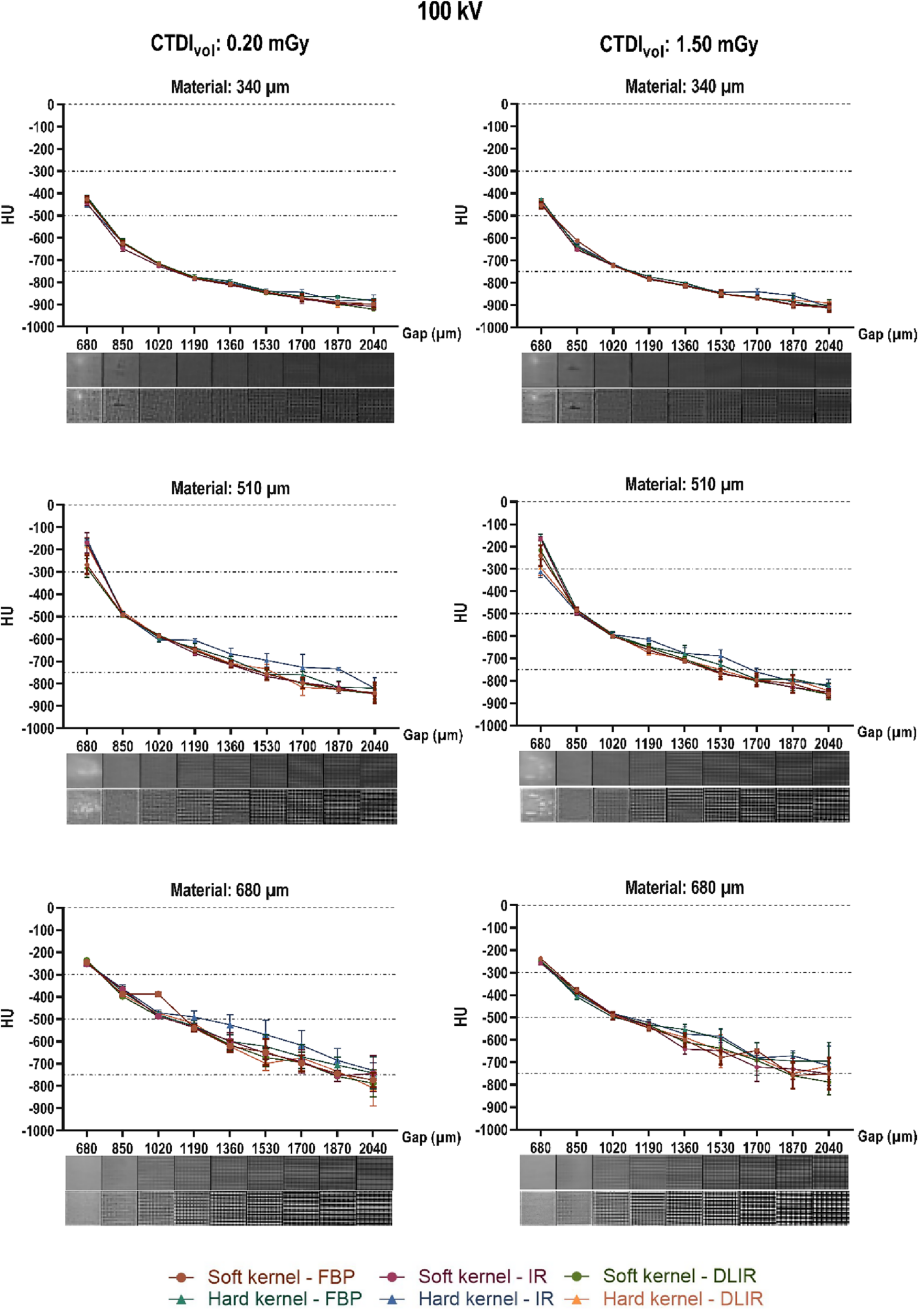
Measured HU values as a function of different three-dimensional-printing settings at 100 kV. Measured HU values are displayed as a function of the designed void side length (in µm) for each of the three material thicknesses (340, 510, and 680 µm) at a tube potential of 100 kV. Each symbol on the curve represents the average HU from triplicate measurements on a CT image acquired at a tube potential of 100 kV, with a computed tomography dose index of either 0.20 mGy (left) or 1.50 mGy (right), that were reconstructed with a specific combination of reconstruction kernels and algorithms. The dotted lines depict the target HU value ranges of the patient-specific radiodensities. The pictures below each graph show each cube printed with a combination of the corresponding gap side length and resin thickness on images reconstructed with DLIR in combination with a soft kernel (top row of pictures) and a hard kernel (bottom row of pictures). CTDI_vol_, Computed tomography dose index volume; DLIR, Deep-learning image reconstruction; FBP, Filtered back projection; IR, Iterative reconstruction

HU measurements of each of the cubes for CT image acquisitions at 100 kV and with varying reconstruction settings are presented in Fig. 5 as the mean of triplicate measurements with their according standard deviation. Supplementary Fig. S1 shows analogous measurements for image acquisitions of the cubes at 120 kV and 140 kV, reconstructed with the same combinations of kernels and algorithms as seen in Fig. 5. A broad range of HU values was attained from -915 to a maximum of -141. Overall, measured radiodensities showed no major differences across different CT settings.

From a certain void side length threshold, for almost all cubic samples, the grid structure became visible on the CT images. For the smaller filament thickness of 340 µm, this discernible lattice structure was mostly visible on images reconstructed with a hard kernel and only for the structures with void side lengths from 1,700 µm. However, for the material printed in a wider thickness, especially the 680 µm, on both the soft and the hard kernel, the print pattern became visible as soon as the designed void side length is 1,020 µm or more. This is shown in Fig. 5, where the pictures below the ticks on each *x*-axis show the cube printed with a combination of the according gap side length and the resin thickness. For each of the two rows of pictures, the upper ones are images of the cubes reconstructed with DLIR in combination with the soft kernel, whereas the lower ones are images reconstructed with DLIR and a hard kernel. CT images from the other combinations of reconstruction algorithms with both kernels and the other tube potentials (Fig. S1, 120 and 140 kV) display the same behaviour and are omitted for visualisation purposes.

The visible lattice structure also causes large CT value standard deviations, as the variable air-resin proportion results in varying the average measured HU number. This is caused by the fact that in the region of interest used to measure the HU values, some pixels have a CT number closer to the resin while others have numbers closer to air, creating large standard deviations. Besides, in some cases, large inhomogeneities in the material were observed in the cubic structure (Fig. 5). These inhomogeneous material distributions were visible in all combinations of the resin printed with a thickness of 340 and 510 µm with a void side length of 680 µm. In all cases of 340 µm material thickness and gaps with a side length of 850 µm, there could be ‘air gaps’ observed where the resin appeared to be absent.

### Design and fabrication of part-solid pulmonary nodules

**Fig. 6:**
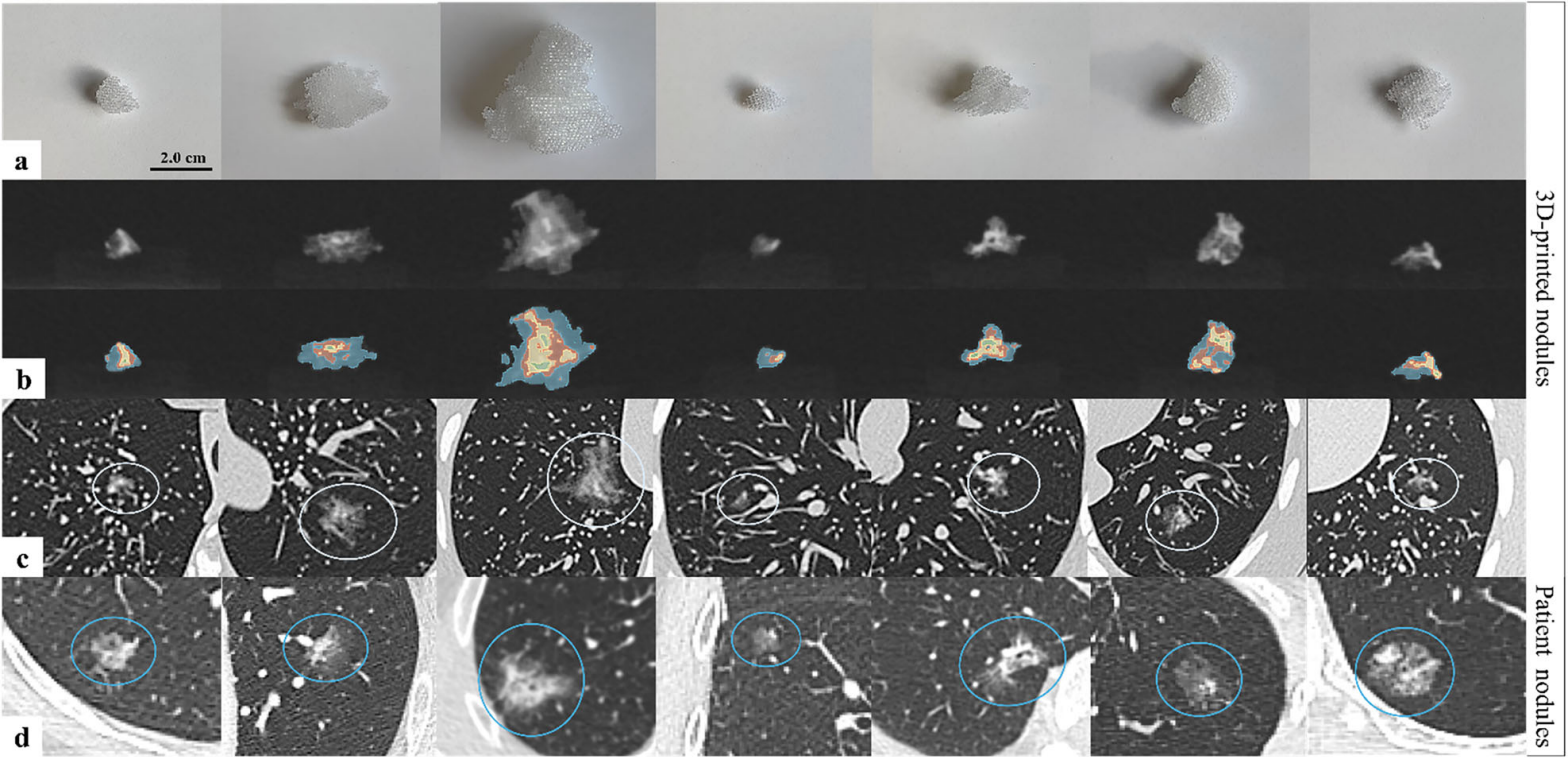
Overview of the 3D-printed part-solid pulmonary nodules. **a** Photograph of the 3D-prints. **b** CT acquisition of the 3D-prints free in air. The top part of the pictures shows the HU value in grayscale, in the lower part the 3D-print is shaded by HU thresholding: green (> 0 HU); yellow (0; -300 HU), red (-300; -500 HU), and blue (-500; -750 HU). **c** CT acquisition of the 3D-prints inside the anthropomorphic Lungman phantom (white circle). **d** Original CT acquisition of the patient nodules on which the design of the 3D-printed nodules was based (blue circle). Consider that it was not the study objective to manufacture 3D-printed nodules that are an exact copy of the patient-derived nodules. 3D*,* Three-dimensional; CT, Computed tomography

Taking into consideration the HU values determined from the patient CT image series and avoiding possible visibility of the 3D-printed grid structure and associated large standard deviations on the average HU values, we selected the 3D-print settings as previously mentioned in Fig. 4 (“Methods”). Realistic design of a part-solid nodule was attempted to be achieved with the creation of three different densities of ground glass. Figure 6 shows photographs and CT image acquisition of the final 3D-prints.

Table 1 summarises design- and cost-related parameters of both the 3D-printed nodule phantoms manufactured according to the reported methodology, as well as a commercially available counterpart. Consider that the information of the latter phantom models is derived from reports and leaflets distributed by the manufacturer [54].

**Table 1:** Summary of characteristics of the 3D-printed part-solid pulmonary nodule phantoms developed with the methodology in this study and the commercial counterpart produced by Kyoto Kagaku

	3D-printed part-solid nodule phantoms	Commercial counterpart
Design		
Material	Resin (Formlabs Clear V4)	Urethane-based resin
Number of densities GGOs	In this study: 3	1
HU ranges of GGOs	(0, -750 HU)	-650
Customisation	High level	None, standardised design options (concentric or eccentric core)
Realism	Patient-based design	Rudimentary design
Complexity	High	Minimal
Nodule edges/margins	Soft/ intricate	Sharp
Costs		
Print material	€160/1,000 mL resin & 6.657 mL resin needed for 1 nodule ± €1/nodule	Set price per set (quote: €5,000, excluding Value Added Tax)
Work/design hours	About 3 h/ nodule (about 50% is computing time for Boolean intersections)	
Reprints	Easily available at the sole cost of printing material	

Costs are estimated for our particular study set-up, having access to an in-house stereolithography three-dimensional printing technique. *GGOs* Ground-glass opacities

### Proof-of-concept demonstration of 3D-printed part-solid pulmonary nodules

The overall accuracy of all scored nodules was 53.5% (95% confidence interval: 49.7−57.5). Importantly, the false negative rate indicates that across all radiologists, 47.5% (44.0−51.0) of the 3D-printed nodules were incorrectly scored and seen as a real patient nodule. All results are shown in the confusion matrices (absolute as well as normalised numbers) in Tables 2 and 3, showing the results respectively for all radiologists combined and divided based on the experience level of the scorer (experienced or in training). The κ coefficient was equal to 0.0495, indicating poor inter-reader agreement.

**Table 2:** Contingency table with results of the single-blinded reader study for all radiologists combined

Overall (17 radiologists)			
		**Computed tomography series (actual)**	
		**3D-printed nodule**	**Patient nodule**
**Scored**	**3D-printed nodule**	375 true positives (0.53)	159 false positives (0.45)
	**Patient nodule**	339 false negatives (0.47)	198 true negatives (0.55)
Overall accuracy		53.5% (49.7−57.5)	
True positive rate		52.5% (49.2−56.0)	
True negative rate		55.5% (46.3−65.9)	
False negative rate		47.5% (44.0−61.0)	
False positive rate		44.5% (34.1−53.7)	

Upper part of the table shows the contingency table with the absolute number of nodules within each class, as well as the normalised number per class (decimal between parentheses). Note that true positives and true negatives are, respectively, 3D-printed nodules and patient nodules that are correctly scored by the radiologists as such. False positives are patient nodules that have been incorrectly determined to be 3D-printed nodules, while false negatives are 3D-printed nodules that have been incorrectly scored as patient nodules by the radiologists. The lower part of the table shows the calculated overall accuracy as well as true/false positive/negative rates. Each value is followed by its 95% confidence interval shown between parentheses. *3D* Three-dimensional

**Table 3:** Contingency tables with results of the single-blinded reader study, divided based on the experience of the radiologists (experienced radiologists and residents in training)

Experienced radiologists (*n* = 5)				Residents in training (*n* = 12)			
		**Computed tomography series (actual)**				**Computed tomography series (actual)**	
		**3D-printed nodule**	**Patient nodule**			**3D-printed nodule**	**Patient nodule**
**Scored**	**3D-printed nodule**	89 true positives (0.42)	51 false positives (0.49)	**Scored**	**3D-printed nodule**	286 true positives (0.57)	108 false positives (0.43)
	**Patient nodule**	121 false negatives (0.58)	54 true negatives (0.51)		**Patient nodule**	218 false negatives (0.43)	144 true negatives (0.57)
Accuracy		45.4% (40.6−50.5)		Accuracy		56.8% (52.1−61.8)	
True positive rate		42.4% (37.3−47.4)		True positive rate		56.8% (52.0−61.5)	
True negative rate		51.4% (41.0−62.2)		True negative rate		57.1% (46.8−68.4)	
False positive rate		48.6% (37.8−59.0)		False positive rate		42.9% (31.6−53.2)	
False negative rate		57.6% (52.6−62.7)		False negative rate		43.3% (38.5−48.0)	

Upper part of the table shows the contingency tables for the two groups of radiologists, divided based on experience level, with the absolute number of nodules within each class as well as the normalised number per class (decimal between parentheses). Note that true positives and true negatives are, respectively, 3D-printed nodules and patient nodules that are correctly scored by the radiologists as such. False positives are patient nodules that have been incorrectly determined to be 3D-printed nodules, while false negatives are 3D-printed nodules that have been incorrectly scored as patient nodules by the radiologists. The lower part of the table shows the calculated overall accuracy as well as true/false positive/negative rates, below each applicable contingency table. Each value is followed by its 95% confidence interval shown between parentheses. *3D* Three-dimensional

The calculated *χ*^2^ goodness of fit test statistic for all radiologists combined shows a value of 136.13. The associated *p*-value of 1.864e-31 indicates that there is, on a 5% confidence level, enough evidence to reject the null hypothesis *H*_0_, which states that the radiologists would be able to distinguish between a real patient subsolid nodule and a 3D-printed model. Moreover, values of *χ*^2^ = 70.0, *p* = 5.930e-17 for senior radiologists and *χ*^2^ = 72.0, *p* = 2.126e-17 for radiology residents in training show no difference in results conditional on the level of experience. While the error rates appear to be distributed slightly differently between the two groups of radiologists, the goodness of fit statistic remains highly significant, conditional on each group.

Generally, reported confidence levels were overall low and varied only modestly between correct and incorrect classifications, suggesting radiologists were generally unsure regardless of the outcome. Full breakdown of confidence levels, likelihood ratios and trends conditional on the reader experience is provided in Supplementary material Section 1, including Table S2.

## Discussion

In this study, we developed an innovative methodology for SLA-type 3D-printing that enabled the printing of structures with varying radiodensities using a single material. Models of part-solid pulmonary nodules were manufactured and found to be indistinguishable from real patient cases through a single-blinded reader study as proof-of-concept demonstration. This methodology allows researchers to tailor phantom properties to specific research requirements or patient-specific scenarios more cost-effectively and efficiently than with current generic phantoms.

Recent increase in low-dose CT research has been accompanied by increased use of anthropomorphic thorax phantoms, with the Lungman phantom being the most commonly used [4, 13, 33–42, 48, 55]. Nevertheless, the pure ground-glass and part-solid nodules proposed by Kyoto Kagaku as an addition to the Lungman phantom inadequately represent complex lung lesions with heterogeneous density and irregular shapes and edges [54]. These generic nodule models comprise one or two dense cores with equal density, surrounded by a less dense outer sphere without smooth transitions between contrast levels or any spiculated or lobulated edges and can cost up to € 5,000. As such, we have manufactured part-solid pulmonary nodule phantoms at less than half the price of existing alternatives. Although we did not dispose of these commercially available nodules to compare directly, we could compare the characteristics, as reported by the manufacturer, with the properties of our 3D-printed models. The information in Table 1 suggests that we produced realistic phantoms with intricate, lifelike anatomy and complex radiological properties on CT images. The true cost-effectiveness of our developed methodology lies in its relative affordability and customisation potential beyond a standardised design. As such, with the increasing availability of 3D-printing, this method enables repeated, personalised phantom manufacturing, with printing material as the only recurring expense.

The recent study of Hatamikia et al [3], based on FDM-printing, reported to be the first study to develop a method to create heterogeneous tumour phantoms with realistic anatomy. Because the purchase of new FDM 3D-printers and filaments would beat the purpose of creating inexpensive phantoms in a fast manner, we adapted their proposed method to our in-house SLA-printing technology. Intrinsically, SLA provides higher resolution prints than FDM [14, 17]. Additionally, we enhanced the design method by including more than two radiodensities within one nodule as opposed to the study of Hatamikia et al [3]. Based on current information, this is the first study to create a phantom using SLA and a single resin to print multiple densities with smooth transitions between the different contrast levels.

The rapid advancements in 3D-printing allowed research groups across various medical disciplines to manufacture test objects tailored to their specific need [3–8, 11–18, 23]. Despite increasing realism and customisation of the 3D-printed phantoms, their clinical relevance remains a subject of discussion. Most studies either do not evaluate or only provide an approximate assessment of the phantom’s applicability in later research [3, 4, 6, 8, 10, 12, 13, 16, 18]. Therefore, we subjected the final 3D-prints to validation with a vast cohort of radiologists. Using a single-blinded distinguishability test, we could objectively assess whether the 3D-printed phantom nodules resembled clinical examples of part-solid pulmonary nodules. The *χ*^2^ goodness of fit test results and the inter-reader *κ* coefficient of 0.0495 both suggest that the classifications by the readers were mere random guesses [53]. This demonstrates that our 3D-printed part-solid nodules cannot be reliably differentiated from real part-solid nodules in patients, highlighting their ability to replicate highly intricate morphologies with multiple heterogeneities. Although we do appreciate that some clinical applications or routine tasks are feasible with simple instead of (hyper)-realistic phantoms, the authors emphasise that an objective validation tailored to the intended research purpose, and its associated degree of required complexity, is essential.

The realistic morphology and characteristic gradients of densities are imperative for pulmonary nodule research. Particularly, validation studies of computer-aided detection and volumetry algorithms benefit from the use of realistic phantom setups. Our previous research applied the anthropomorphic Lungman phantom containing only solid, well-defined nodules 3D-printed fully dense in the same resin (Formlabs Clear V4) [33]. However, a limitation of this study was the insufficient representation of clinically detected nodules [33]. Therefore, our current proposed workflow enables us to build upon previous studies by incorporating results from phantom setups that more accurately reflect the natural course of lung cancer. In addition to part-solid nodules, we propose that this method can be adjusted to manufacture other appearances of pulmonary nodules [56]. By, for example, printing the different lattice structures as proposed in this workflow, but leaving out the fully dense core, a subsolid nodule model could be constructed. Deliberate inclusion of larger air gaps in the lattice structures could potentially replicate bubble-like lucencies. Also, resins with higher radiological attenuation might be applicable in modelling lesion calcifications.

There are numerous potential applications where adaptation of our phantom nodules could be of value. Phantom models provide an inherent absolute ground truth, essential in training and validation of artificial intelligence-based detection and volumetric algorithms. In medical physics, assessment of technical image quality parameters, like noise and contrast resolution, on CT images of human-like nodular structures can guide low-dose scanning protocol optimisation or help shape the development of required phantoms for research and daily quality assurance in lung cancer screening.

A potentially interesting application would be in the field of radiomics. The main limitation of radiomics is the low robustness of extracted radiomic features with respect to variabilities in CT scanner or protocol-specific acquisition and reconstruction parameters, and as such, potentially limiting the performance of predictive models built upon them [57, 58]. Our phantom models could provide a standardised setup with similarities to real human nodule structures to test and correct the dependence of radiomic features on exposure settings, such as the tube potential and tube current, or reconstruction parameters, such as the algorithm or kernel [57–60]. Regardless, we anticipate that research groups will be able to customise and adapt our published method to suit their specific needs and available materials. Furthermore, as this method allows for the gradual decrease of the radiological density of the print material used in SLA 3D-printing, it could theoretically be applied to model other structures that show low differences in contrast on CT images. Consider, for example, phantoms of the abdomen or the middle mediastinum, which contain organs and structures that cover a small HU window.

Even though technical information of our nodule models is open-sourced (see stereolithography files, electronic supplementary material), we appreciate that this method is purposefully developed for a specific application. Firstly, it is crucial to conduct an initial evaluation and optimisation. Reports indicate that the density of a printed object, and consequently its radiological characteristics, depends not only on the type of material but also on the specific printer used [17, 29]. We presume that adaptation of a different resin might require different combinations of material thickness and void sizes to achieve the same HU ranges as observed in this study. The applied post-processing steps, such as curing and washing, might also influence radiological densities of the 3D-prints, and their influence should be further investigated.

Moreover, an evaluation of the 3D-printing reproducibility on the same printer needs to be performed, as currently only one sample per design was printed. The same assessment regarding reproducibility across independent SLA-printers should be conducted. Secondly, on some image acquisitions reconstructed with a hard kernel, the grid structure was still visible. The perceptible spaces may result from contractions occurring after the cooling down of the printing material [10, 23, 24, 30].

Although the data indicate that no single nodule was consistently recognised as a 3D-printed phantom beyond a random guess, the possibility of task ambiguity or reader confusion in the binary response design of the current proof-of-concept demonstration cannot be ruled out, and further clinical validation could be implemented. Also, radiological properties can still vary based on the adapted CT system or protocol parameters. However, depending on the characteristic density of the resin, some printing materials might be restricted in the decrease in ranges of HU values they can reach, while maintaining realism and homogeneity on the CT images. Lastly, while our methodology allows us to create phantom models that have the potential to be of lower cost than their commercially available counterparts, the validation of the 3D-printed nodules in this study still included a comparatively expensive generic Lungman phantom.

In conclusion, we developed a workflow for designing, fabricating, and validating SLA 3D-printed nodule phantoms with patient-realistic morphological and radiological properties. This approach allowed us to produce clinically relevant part-solid pulmonary nodule models that are customisable, relatively inexpensive and quickly available as opposed to generic, commercial alternatives. Our findings highlight the potential for research groups to expand and refine the use of 3D-printing for manufacturing phantom models, supporting various processes for optimisation and validation of CT imaging techniques.

## Supplementary information

**Additional file 1: Table S1.** Specification of the patient-specific part-solid nodules. **Fig. S1.** Measured HU values in function of different three-dimensional printing settings (120 and 140 kV). Measured HU values in function of the designed void side length (in µm) for each of the three material thicknesses (340, 510, and 680 µm) at a tube potential of 120 kV or 140 kV. Each symbol on the curve represents the average HU from triplicate measurements on CT image acquired at a tube potential of 120 kV or 140 kV, with a computed tomography dose index of either 0.20 mGy or 1.50 mGy, which was reconstructed with a specific combination of reconstruction kernels and algorithms. The dotted lines depict the target HU value ranges of the patient-specific radiodensities. **Table S2.** Frequency and likelihood ratios of the degree of confidence reported in the single-blinded reader study.

Supplemenatry material 1

Supplemenatry material 2

Supplemenatry material 3

Supplemenatry material 4

Supplemenatry material 5

Supplemenatry material 6

Supplemenatry material 7

Supplemenatry material 8

Supplemenatry material 9

Supplemenatry material 10

## Data Availability

The stereolithography files (.stl) with the final designs of the lattice structures and complete part-solid nodule models are included as supplementary electronic files within the article. Other technical files or datasets used and/or analysed during the current study are available from the corresponding author on reasonable request.

## Abbreviations

CT: Computed tomography
DLIR: Deep-learning image reconstruction
FDM: Fused deposition modelling
IR: Iterative reconstruction
SLA: Stereolithography apparatus
