# Supplementary material for: Part-solid pulmonary nodule phantoms with realistic morphology and densities by stereolithography-based 3D-printing: from design to validation
Source: Eur Radiol Exp. 2025 Nov 4;9:106. doi: 10.1186/s41747-025-00644-4 (PMC12586819; doi:10.1186/s41747-025-00644-4)
Supplement: Supplementary file 1 — Additional file 1: Table S1. Specification of the patient-specific part-solid nodules. Fig. S1. Measured HU values in function of different three-dimensional printing settings (120 and 140 kV). Measured HU values in function of the designed void side length (in µm) for each of the three material thicknesses (340, 510, and 680 µm) at a tube potential of 120 kV or 140 kV. Each symbol on the curve represents the average HU from triplicate measurements on CT image acquired at a tube potential of 120 kV or 140 kV, with a computed tomography dose index of either 0.20 mGy or 1.50 mGy, which was reconstructed with a specific combination of reconstruction kernels and algorithms. The dotted lines depict the target HU value ranges of the patient-specific radiodensities. Table S2. Frequency and likelihood ratios of the degree of confidence reported in the single-blinded reader study. [file 41747_2025_644_MOESM1_ESM.pdf]

Part-solid pulmonary nodule phantoms with realistic morphology and densities by stereolithography-based 3D printing: from design to validation

ELECTRONIC SUPPLEMENTARY MATERIAL

Table S1 Specification of the patient-specific part-solid nodules

Patient Nodule	CT scanner parameters			Nodule measurements - subsolid			Nodule measurements - solid		
	CT scanner	kVp	Reconstruction algorithm & kernel	HU value	Min	Max	HU value	Min	Max
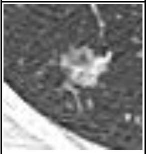	GE LightSpeed VCT	120	IR & Lung	-576.3<br>-560.3<br>-423.3<br>Mean: -519.9<br>StDev: 84.1	-677<br>-639<br>-472	-468<br>-443<br>-340	96.0<br>46.8<br>15.8<br>Mean: 52.8<br>StDev: 40.5	-90<br>-12<br>-86	225<br>153<br>81
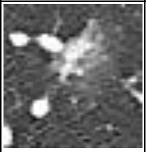	GE LightSpeed VCT	120	IR & Lung	-433.8<br>-626.5<br>-601.8<br>Mean: -554.0<br>StDev: 104.9	-485<br>-661<br>-649	-411<br>-593<br>-568	93.8<br>72.5<br>88.8<br>Mean: 85.0<br>StDev: 11.1	2<br>5<br>-7	148<br>147<br>176
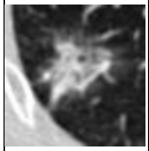	Siemens Biograph Vision	100	IR & B10f/ very smooth	-289.5<br>-369.5<br>-465.8<br>Mean: -374.9	-328<br>-415	-256<br>-325	174.8<br>248.3<br>109.8<br>Mean: 177.6	157<br>174<br>84	193<br>336<br>138

				StDev: 88.3	- 499	- 425	StDev: 69.3		
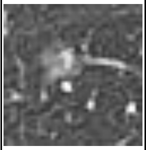	GE Revolution	100	IR & Lung	-541.0<br>-543.5<br>-550.5<br>Mean: -545.0<br>StDev: 4.9	- 608<br>- 591<br>- 608	- 478<br>- 500<br>- 505	2.3<br>188.5<br>55.0<br>Mean: 81.9<br>StDev: 96.0	- 209<br>58<br>- 247	196<br>339<br>261
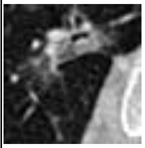	Siemens Sensation 16	120	IR & B50f/ medium sharp	-373.5<br>-515.0<br>-434.8<br>Mean: -441.1<br>StDev: 71.0	- 404<br>- 562<br>- 502	- 331<br>- 480<br>- 341	356.8<br>140.8<br>270.5<br>Mean: 256.0<br>StDev: 108.7	317<br>45<br>247	433<br>214<br>293
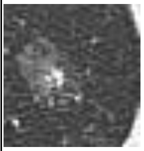	GE LightSpeed VCT	120	IR & Lung	-637.8<br>-572.5<br>-694.5<br>Mean: -634.9<br>StDev: 61.1	- 694<br>- 589<br>- 750	- 587<br>- 562<br>- 632	103.0<br>256.5<br>82.3<br>Mean: 147.3<br>StDev: 95.2	-53<br>65<br>-99	196<br>577<br>219
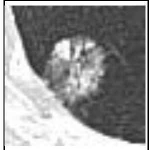	GE LightSpeed VCT	120	IR & Lung	-412.0<br>-532.8<br>-529.5<br>Mean: -491.4<br>StDev: 68.8	- 454<br>- 612<br>- 666	- 380<br>- 486<br>- 393	74.0<br>111.8<br>93.3<br>Mean: 93.0<br>StDev: 18.9	33<br>2<br>32	141<br>233<br>146

For each patient, acquisition and reconstruction parameters of their CT scan is given. In addition, the HU values (measured value, minimum and maximum) of the subsolid and solid component of the part-solid nodules were recorded in Fiji and are presented together with the calculated mean HU and its standard deviation. CT Computed tomography, *IR* Iterative reconstruction, *StDev* Standard deviation.

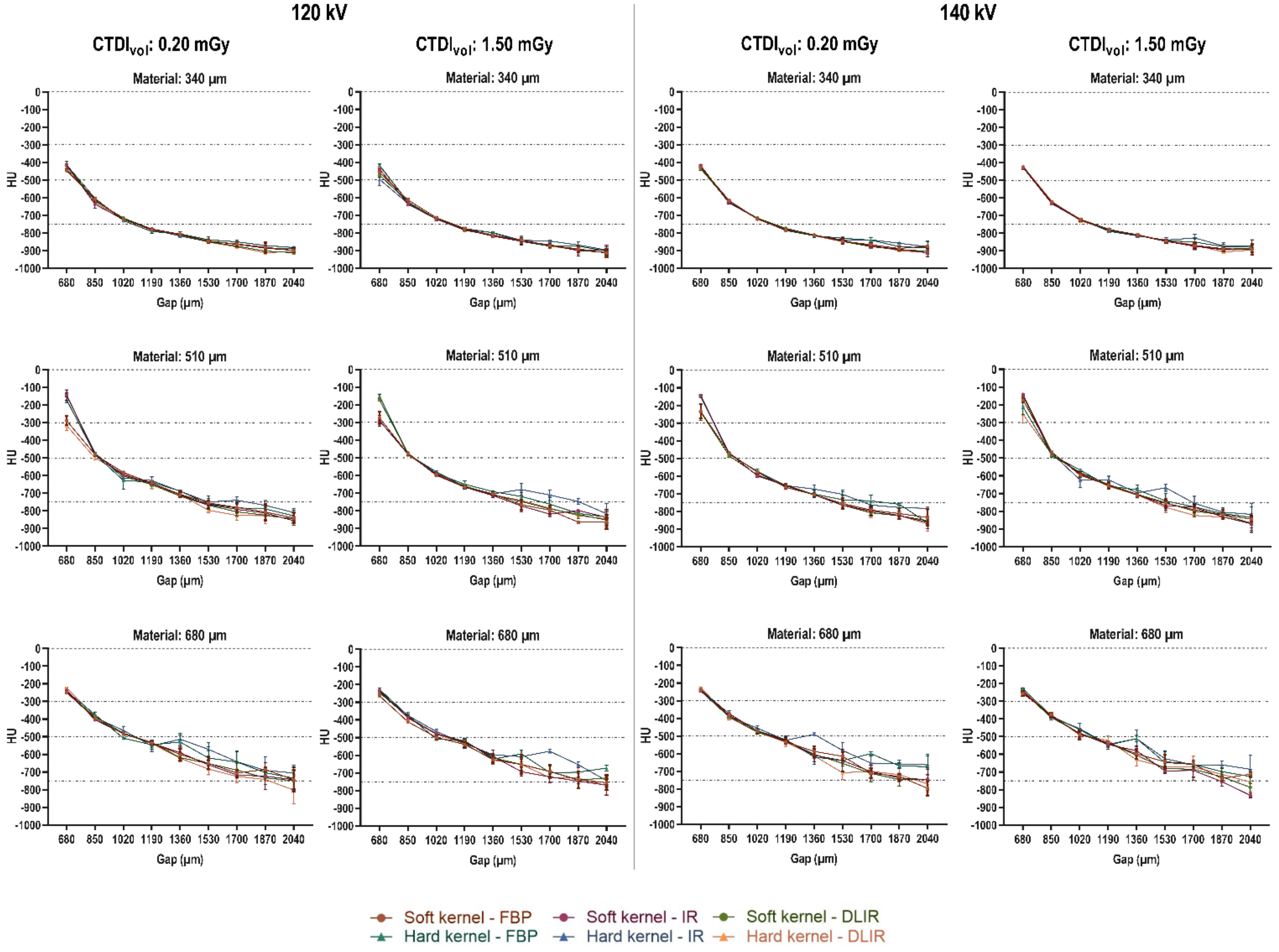

Fig. S1 Measured HU values in function of different three-dimensional printing settings (120 and 140 kV). Measured HU values in function of the designed void side length (in µm) for each of the three material thicknesses (340, 510 and 680 µm) at a tube potential of 120 kV or 140 kV. Each symbol on the curve represents the average HU from triplicate measurements on CT image acquired at a tube potential of 120 kV or 140 kV, with a computed tomography dose index of either 0.20 mGy or 1.50 mGy, which was reconstructed with a specific combination of reconstruction kernels and algorithms. The dotted lines depict the target HU value ranges of the patient-specific radiodensities. *$CTDI_{vol}$* Computed tomography dose index volume, *DLIR* Deep learning image reconstruction, *FBP* Filtered back projection, *IR* Iterative reconstruction.

Full breakdown of the reported confidence levels

In each of the cases where the classification of the presented nodule was either correct (True positives and negatives) or incorrect (False positives and negatives), we looked at the according level of confidence that was given with this answer and results are presented in Table 4. This shows that overall confidence of all answer given was rather low and most answers were given with at most some confidence. Some trends in the level of experience can be observed based on the level of experience of the radiologists. Table 4 also includes likelihood ratios, both within a group of readers as well as between the groups with differing experience. Conditionally whether classification was correct or incorrect, this displays how much more (> 1) or less (< 1) likely the level of confidence is to be reported or if there is no difference (= 1). For the more experienced radiologists, for almost all degrees of confidence, there is no remarkable difference in the frequency whether or not the presented nodule was correctly classified. The likelihood for the highest level of confidence is observed to be somewhat higher when the experienced radiologists scored initially correct. For the radiologists in training, it is observed that the frequency of higher reported confidence levels increased up to double the likelihood when the nodule was scored correctly. Vice versa, they were half as likely to report no confidence when they were correct. Overall, the group of more experienced radiologists appears to have a more extreme reported level of confidence (either no confidence or completely confident), while the less experienced readers are more moderate in their level of confidence and are showing to be more likely to report some confidence. This trend appears to be irrespective of the fact that the initial answer of the radiologists was correct or incorrect. As such, this suggests that radiologists were about equally (un)certain, whether they gave a correct or incorrect answer.

Table S2 Frequency and likelihood ratios of the degree of confidence reported in the single-blinded reader study

Overall (17 radiologists)						
		Frequency of answer (%)				
		Not at all confident	Slightly confident	Somewhat confident	Fairly confident	Completely confident
Scored	Correct	6.8	28.5	33.7	27.6	3.5
	Incorrect	10.1	31.6	39.6	16.5	2.2

Experienced radiologists (*n* = 5)						
		Frequency of answer (%)				
		Not at all confident	Slightly confident	Somewhat confident	Fairly confident	Completely confident
Scored	Correct	12.6	37.1	30.1	15.4	4.9
	Incorrect	12.8	35.5	34.3	14.0	3.5
		Likelihood of confidence				
	$\frac{\text{Frequency of answer} \mid \text{Correct}}{\text{Frequency of answer} \mid \text{Incorrect}}$	1.0	1.0	0.9	1.1	1.4

Residents in training (*n* = 12)						
		Frequency of answer (%)				
		Not at all confident	Slightly confident	Somewhat confident	Fairly confident	Completely confident
Scored	Correct	4.9	25.6	34.9	31.6	3.0
	Incorrect	8.6	29.5	42.5	17.9	1.5
		Likelihood of confidence				
	$\frac{\text{Frequency of answer} \mid \text{Correct}}{\text{Frequency of answer} \mid \text{Incorrect}}$	0.6	0.9	0.8	1.8	2.0

Experienced radiologists >< Residents in training						
		Likelihood of confidence				
Scored correct	$\frac{\text{Frequency of answer} \mid \text{Experienced}}{\text{Frequency of answer} \mid \text{In training}}$	2.6	1.5	0.9	0.5	1.6

Scored incorrect	$\frac{\text{Frequency of answer} \mid \text{Experienced}}{\text{Frequency of answer} \mid \text{In training}}$	1.5	1.2	0.8	0.8	2.3

Frequencies of the reported degree of confidence given in case the answered classification of the presented nodule (real patient nodule versus 3D-printed nodule) was either correct or incorrect for all radiologists combined and divided based on experience level (experienced radiologists and residents in training). For both groups separately, the likelihood to report each of the levels of confidence is shown, conditionally on the classification of the presented nodule. Bottom part of the table displays how much more (> 1) or less (< 1) likely the experienced radiologists were observed to report a particular level of confidence, conditionally on the accuracy (scored correct or incorrect) of the presented nodules.

Stereolithography electronic files

Supplementary material 1_Lattice structure 340-1020.stl

Supplementary material 2_Lattice structure 510-850.stl

Supplementary material 3_Lattice structure 680-680.stl

Supplementary material 4_Nodule 1.stl

Supplementary material 5_Nodule 2.stl

Supplementary material 6_Nodule 3.stl

Supplementary material 7_Nodule 4.stl

Supplementary material 8_Nodule 5.stl

Supplementary material 9_Nodule 6.stl

Supplementary material 10_Nodule 7.stl
